# Patterns and predictors of adherence to health-protective measures during COVID-19 pandemic in the UK: cross-sectional and longitudinal findings from the HEBECO study

**DOI:** 10.1186/s12889-022-14509-7

**Published:** 2022-12-14

**Authors:** Dimitra Kale, Aleksandra Herbec, Emma Beard, Natalie Gold, Lion Shahab

**Affiliations:** 1 Department of Behavioural Science and Health, University College London, London, UK; 2 SPECTRUM Consortium, London, UK; 3 Department of Clinical, Educational and Health Psychology, UCL, London, UK; 4 Institute–European Observatory of Health Inequalities, Calisia University, Kalisz, Poland; 5 Behavioural Insights, Public Health England, London, UK

**Keywords:** Adherence, Health-protective measures, COVID-19, UK, Behaviour, Wash hands, Wear masks, Physical distance, Disinfectant

## Abstract

**Background:**

Adherence to health-protective behaviours (regularly washing hands, wearing masks indoors, maintaining physical distancing, carrying disinfectant) remains paramount for the successful control of COVID-19 at population level. It is therefore important to monitor adherence and to identify factors associated with it. This study assessed: 1) rates of adherence, to key COVID-19 health-protective behaviours and 2) the socio-demographic, health and COVID-19-related factors associated with adherence.

**Methods:**

Data were collected on a sample of UK-based adults during August–September 2020 (*n* = 1,969; lockdown restrictions were eased in the UK; period 1) and November 2020- January 2021 (*n* = 1944; second UK lockdown; period 2).

**Results:**

Adherence ranged between 50–95%, with higher adherence during the period of stricter measures. Highest adherence was observed for wearing masks indoors (period 1: 80.2%, 95%CI 78.4%-82.0%, period 2: 92.4%, 95%CI 91.1%-93.6%) and lowest for carrying own disinfectant (period 1: 48.4%, 95%CI 46.2%-50.7%, period 2: 50.7%, 95%CI 48.4%-53.0%). Generalized estimating equation models indicated that key factors of greater odds of adherence included being female, older age, having higher income, residing in England, living with vulnerable individuals and perceived high risk of COVID-19.

**Conclusions:**

Targeted messages to different demographic groups may enhance adherence to health-protective behaviours, which is paramount for the control of airborne respiratory diseases.

**Protocol and analysis plan Registration:**

The analysis plan was pre-registered, and it is available at https://osf.io/6tnc9/.

**Supplementary Information:**

The online version contains supplementary material available at 10.1186/s12889-022-14509-7.

## Introduction

The rapid spread and significant mortality rate of COVID-19 prompted governments worldwide to introduce temporary lockdowns and a range of behavioural guidelines to tackle the COVID-19 pandemic, such as wearing facemasks, maintaining physical distance to others when in public, and washing hands regularly (i.e., on 9th September 2020 these three protective measures were promoted in the UK using a campaign: “HANDS, FACE, SPACE” [1]). Existing evidence suggests that such measures can reduce the transmission of the virus and consequently reduce overall mortality [2, 3]. However, the success of this approach depends largely on the level of adherence to these measures. Comprehensive vaccination programmes are in place in most countries such as the UK, and although they offer protection against COVID-19 adherence to health-protective behaviours remains paramount for the successful control of virus transmission at population level.

Health psychology models of behaviour would suggest that those who have (and/or perceive) a greater risk of infection with the SARS-CoV-2 virus, or who suffer more severe consequences of COVID-19, should adhere more to health-protective behaviours (e.g., [4]). Those who are at increased risk of COVID-19 include key workers (e.g., healthcare professionals, cashiers in supermarkets) that are unable to carry out their work from home and are therefore more exposed to potentially infected people [5]. Further, specific characteristics predispose some to a higher risk of a severe disease course, e.g., being overweight or obese, from black and minority communities, or having a pre-existing medical condition [5]. Some health behaviours also theoretically increase risks of COVID-19 infection, such as smoking due to regular hand-to-mouth movement [6]. It has been suggested that certain social and economic determinants may make it easier for individuals to adhere to social distancing and enhanced hygiene guidelines. For example, those with higher education and income may be in a better position to access and comply with the recommendations, including having masks or being able to distance themselves, such as working from home. The level of adherence could also depend on local regulations across countries (e.g., different local regulations apply to constituent countries of the UK, [7]).

Preliminary evidence suggests that a range of factors may be related to adherence with protective measures during the COVID-19 pandemic and that adherence levels vary depending on the specific behaviour [8, 9]. For instance, while some studies found that low adherence is associated with being younger [10–12] and male [10, 13], perceived low risk of COVID-19 [14] and having riskier attitudes [15], others found no such associations [13, 16]. It was also found that adherence to wearing a facemask was lower than for social distancing measures, at least at the beginning of COVID-19 pandemic [8, 9]. Furthermore, adherence may be dependent on the stage of the pandemic, with higher levels earlier on but lower levels during later stages, either due to depletion or adherence fatigue setting in, or because of mixed messaging on protective behaviours during intermediate periods of relaxation of lockdown measures [9, 17]. However, most of the available studies were conducted in the early days of the pandemic that were often marked by the strictest social distancing measures and more uncertainty about the virus and its transmission. Indeed, the findings from current and previous pandemics (e.g., H1N1) suggest that levels and predictors of adherence do not remain stable across pandemics [18–20]. For example, it has been reported that there was an increase in adherence in wearing facemasks over time in the current pandemic [9], in part due to lack of availability of personal protective equipment earlier on in the pandemic and because wearing a mask was not mandatory in the first months of the pandemic in many countries [21]. These findings suggest that existing COVID-19 studies that focused on data from the early months of the pandemic [13, 15, 22] may not be able to generalize to later stages of the pandemic.

Therefore, this study aimed to assess the level and stability of adherence, as well as the factors associated with adherence, to the key COVID-19 protective measures: regularly washing hands, wearing masks indoors (e.g., in a shop), maintaining the recommended physical distance from the others, as well as carrying own disinfectant (enhanced hygiene), while leaving the house. The first three health-protective behaviours were promoted by the UK Government and will be considered as government-mandated health-protective behaviours in this study. The study covers two periods in the UK: summer 2020 when the 1^st^ lockdown was eased, and less stringent measures were in place (period 1; August–September 2020) and winter 2020 when measures were reintroduced during the 2^nd^ lockdown (period 2; between November 2020-January 2021).

Specifically, the study aimed to address the following research questions (RQs):

- RQ1. What was the level of overall adherence to all government-mandated health-protective behaviours (regularly washing hands, wearing masks indoors, maintaining the recommended physical distance from others; primary aim) as well as adherence to four individual health-protective behaviours (the above three plus carrying own disinfectant; secondary aim) during summer 2020 (period 1; August–September 2020) and winter 2020 (period 2; November 2020-January 2021) in UK?
- RQ2. Which factors (sociodemographic, COVID-19-related, health behaviours and conditions) were associated with overall adherence to government-mandated health-protective behaviours (primary aim) as well as adherence to the individual health-protective behaviours (secondary aim) for the two time periods?
- RQ3. Was there a difference in (i) overall adherence to government-mandated health-protective behaviours (primary aim) or (ii) adherence to the individual health-protective behaviours (secondary aim) between the two periods, and which, if any, factors were associated with this difference?

## Methods

### Study design

**Fig. 1:**
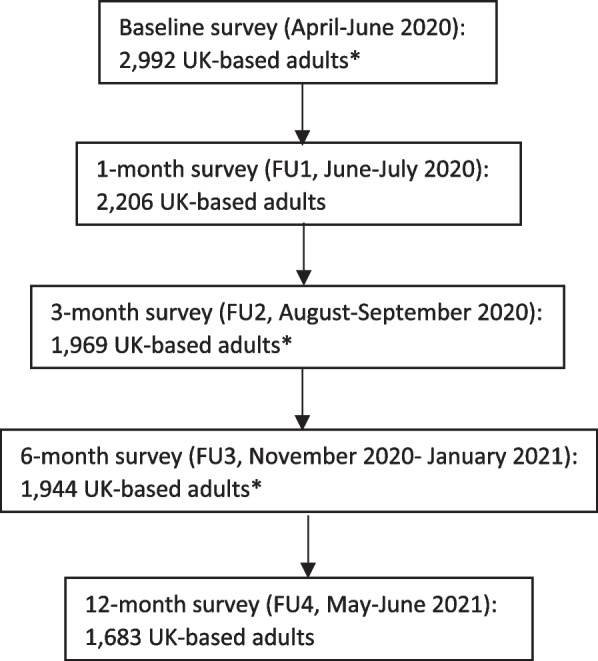
Flowchart of HEBECO research process. *Data collected at these waves were included in the present analysis

Analysis of cross-sectional and longitudinal data from a longitudinal online survey of adults; the Health Behaviours during the COVID-19 pandemic (HEBECO) study (https://osf.io/sbgru/). There have been 5 waves of data collection. Baseline data collection occurred between April and June 2020 and follow-up surveys were administered at 1 month (FU1, June-July 2020), 3 months (FU2, August–September 2020), 6 months (FU3, November 2020- January 2021) and 12 months (FU4, May–June 2021) from the baseline participation date (Fig. 1). This study uses time invariant predictors measured at baseline and time varying predictors measured at FU2 and FU3. The outcome was measured at FU2 and FU3. Data collected at FU1 and FU4 were excluded from the present analysis, as we did not collect data on outcome variables at FU1 and the follow-up rate was low at FU4 (~ 55%).

### Study sample

A self-selected sample of UK-based adults (18 +), fluent in English and willing to complete the baseline and follow-up surveys were recruited online. To increase sample representativeness, the recruitment campaign into the HEBECO baseline involved sharing study materials and invitations via multiple channels, including unpaid and paid advertisements on social media (e.g., Facebook, Twitter, Reddit), email campaign across the network of University College London, other universities, Public Health England, Cancer Research UK, charities, and local authorities across the UK. For full details on the recruitment strategies, visit https://osf.io/sbgru/.

Participants gave their written consent prior to data collection. Data were captured and managed within the REDCap electronic data system [23, 24]. Participants were followed up via email (except for participants who explicitly opted out), with up to three reminders to complete the survey sent at each follow up. Reasons for not completing the follow-up surveys were not assessed.

The study involves cross-sectionally and longitudinally data from UK-based adult participants (18 +), fluent in English and willing to complete the baseline and follow-up surveys and who were not in total isolation at FU2 (period 1) or at FU3 (period 2), i.e., who report leaving the house for any allowed reason, such as to shop, to exercise, to get medications, or to work. Participants who were in total isolation at FU2 or FU3 (i.e., not leaving the house for any reason) were not asked questions about their behaviours when leaving the house and therefore were excluded.

#### Cross-sectional analysis for period 1

Data from UK-based participants who completed the measures of interest as part of FU2 questionnaire (August–September 2020; lockdown restrictions were eased in the UK).

#### Cross-sectional analysis for period 2

Data from UK-based participants who completed the measures of interest as part of FU3 questionnaire (November 2020-January 2021; second UK lockdown and subject to regional differences in restriction severity).

#### Longitudinal analysis

Data from UK-based participants who completed both the FU2 and FU3 questionnaires.

### Measures

All measures were self-reported.

### Outcome measures assessed at FU2 and FU3

Adherence to health-protective behaviours assessed with the question ‘When you leave the house, do you do any of the below?’ The list of behaviours included (i) regularly wash hands, (ii) wear a mask indoors (e.g., shops), (iii) try to keep recommended physical distance to others, (iv) carry own disinfectant. The answer options for each behaviour were: never/rarely/sometimes/often/always at FU2 and never/rarely/often/always at (due to an administrative error the answer option ‘sometimes’ was removed from FU3).

Adherence to individual health-protective behaviours (i, ii, iii, iv) was conceptualised as ‘always’ endorsing the behaviour (coded as 1; [25]) whereas non-adherence was conceptualised as behaviour endorsed less often than ‘always’, including ‘never’, ‘rarely’, ‘sometimes’ or ‘often’ response choices (coded as 0). Overall adherence to government-mandated health-protective behaviours was conceptualised as summing adherence, as defined above, of the three health-protective behaviours (i, ii, iii) that were promoted in the UK [1], resulting in a score ranging from 0 (no adherence) to 3 (perfect adherence). Overall adherence was the primary outcome and adherence to individual health-protective behaviours the secondary outcome.

### Predictors of adherence

Time-invariant predictors assessed at baseline included age (continuous in years), gender (female vs all other), education (post-16 qualification vs not), ethnicity (any white vs all other including prefer not to say), country of residence (England vs other UK countries; Scotland, Wales, Northern Ireland), health condition (no vs yes /prefer not to say), being a keyworker (yes vs no including non-employed, students and retired).

Time-varying predictors assessed at FU2 and FU3 included household income (≥ £50,000 vs < £50,000 vs prefer not to say), perceived COVID-19 risk to one’s health (major/significant risk vs moderate/minor/no risk, and don’t know), experience of social distancing (assessed with the question “How would you rate your overall experience with social distancing restrictions due to COVID-19” measured on a scale from ‘extremely negative (1)’ to ‘extremely positive (100)’), smoking status (current smokers vs not), BMI (overweight/obese with BMI > 25 vs all other; calculated from self-reported weight in kilograms divided by self-reported height in metres squared), quality of life (assessed with the question “How would you rate the following aspects (quality of living conditions, psychological well-being, social and family relationships measured on a scale from ‘poor (1)’ to ‘excellent (5)’ and conceptualised as an average continuous rating from 1–5 of quality of living conditions, psychological well-being, social and family relationships (1 = poor, 5 = excellent)). For two variables—living alone (yes vs no) and living with people considered to be vulnerable to COVID-19 (e.g., those aged 70 and over, those living with comorbidities; yes vs not) – baseline values were used instead of FU2 data as these were not assessed at that follow-up wave.

We also assessed diagnosed or suspected COVID-19 (measured at FU2 and FU3). Participants were asked whether they had ‘been tested for COVID-19 with a swab test (to check current infection)’ and whether they had ‘been tested for COVID-19 with an antibody/blood test (to check past infection)’ with the response options (i) yes and tested positive at least once, (ii) yes and tested negative every time, (iii) yes and awaiting results (iv) no and (v) prefer not to say for both questions. Participants who reported not having had a positive COVID-19 test were asked “The key symptoms for COVID-19 are high temperature/fever or a new, continuous cough/loss or change to your sense of smell or taste. Do you think you HAVE or HAD COVID-19?” with the answer options (i) I think I have COVID-19, (ii) I think I had COVID-19, (iii) I do not think I have or have or had COVID-19, (iv) don’t know and (v) prefer not to say. All participants reporting ‘yes and tested positive at least once’ to question 1 and/or 2 or who reported thinking they have or had COVID-19 to question 3 were considered as diagnosed/suspected COVID-19 cases, with all other responses as not diagnosed/suspected COVID-19 cases.

### Statistical analysis

Statistical analysis was conducted in SPSS Statistics version 27. The protocol and analysis plan were pre-registered on Open Science Framework (https://osf.io/6tnc9/). Descriptive statistics were calculated to characterise the sample on key demographics and study variables. For cross-sectional analyses the data were weighted to Census and Annual Population Survey mid-year estimates for age, gender, ethnicity, country of living, education and household income to account for the non-random nature of the sample using standard methodology [26]. The analysis used weights trimmed to top 98^th^ percentile to minimise the impact of extremely high weights [27].

### RQ1

#### Cross-sectional analysis

The proportion (and 95% Confidence Interval (CI)) of participants with 0, 1, 2 and 3 score in overall adherence to government-mandated health-protective behaviours (regularly washing hands, wearing masks indoors, maintaining the recommended physical distance from others; primary analysis), and the proportion (and 95% CI) of participants who always adhered to the four individual health-protective behaviours (regularly washing hands, wearing masks indoors, maintaining the recommended physical distance from others, carrying own disinfectant were calculated at period 1 and at period 2. While Wilcoxon rank test was used to compare adherence between the two time periods.

### RQ2

#### Longitudinal analysis

First unadjusted generalized estimating equation (GEE) models were used to assess the association between the predictors listed above (i.e., sociodemographic, COVID-19-related, health behaviours and conditions) and i) the combined score on the overall adherence (0–3) to government-mandated health-protective behaviours (regularly washing hands, wearing masks indoors, maintaining the recommended physical distance from others; primary outcome) and ii) the binary measures of adherence (yes/no) on the individual health-protective behaviours (regularly washing hands, wearing masks indoors, maintaining the recommended physical distance from others, carrying own disinfectant; secondary outcomes).

General linear models were fitted using GEE. The specific working correlation matrix for the binary outcome of adherence (yes/no) was: Y_ij_ ~ Binomial (n, π_ij_) for subject i and measurement j. Log( π_ij_/(1-π_ij_) = β_0_ + Gender_i_β_1_ + Ethnicity_i_β_2_ + Qualifications_i_β_3_ + Residence_i_β_4_ + Keyworker _i_β_5_ + Living alone_ij_β_6_ + Living with vulnerable_i_β_7_ + Income < £50,000_i_β_8_ + Income Prefer not to say_i_β_9_ + Non-smoker_i_β_10_ + Obese_i_β_11_ + No health problem_i_β_12_ + Non high risk COVID-19_i_β_13_ + Not diagnosed COVID-19_i_β_14_ + Age_i_β_15_ + Quality of life_i_β_16_ + Experience social distancing_i_β_17_.

For the continuous outcome: Y_ij_ ~ Normal for subject i and measurement j.

Y_ij_ = β_0_ + Gender_i_β_1_ + Ethnicity_i_β_2_ + Qualifications_i_β_3_ + Residence_i_β_4_ + Keyworker _i_β_5_ + Living alone_ij_β_6_ + Living with vulnerable_ij_β_7_ + Income < £50,000_ij_β_8_ + Income Prefer not to say_ij_β_9_ + Non-smoker_ij_β_10_ + Obese_ij_β_11_ + No health problem_i_β_12_ + Non high risk COVID-19_ij_β_13_ + Not diagnosed COVID-19_ij_β_14_ + Age_i_β_15_ + Quality of life_ij_β_16_ + Experience social distancing_ij_β_17_. Each individual time-varying predictor variable model was then adjusted for a main effect of time and for a time*predictor variable interaction. Fully adjusted GEE models containing all exploratory variables was then computed. The fully adjusted GEE models were assessed for goodness of fit using Quasi-Likelihood under Independence Model Criterion (QIC) [28]. Then, fully adjusted GEE models containing all explanatory variables as well as all significant time*predictor variable interactions from the univariate models with time interactions were computed. Time*predictor variable interactions were retained in the full GEE model if they improved QIC > 2 over the full GEE model without interactions, and the interaction itself remained significant (*p* < 0.05).

### RQ3

#### Longitudinal analysis

A change score in adherence i) for the government-mandated health-protective behaviours (regularly washing hands, wearing masks indoors, maintaining the recommended physical distance from others; primary outcome) and ii) for each of the four health-protective behaviours (regularly washing hands, wearing masks indoors, maintaining the recommended physical distance from others, carrying own disinfectant secondary outcomes) between period 1 and period 2 were computed (increased/decreased adherence, remained always adherent/never adherent), and multinomial logistic regressions were used (never adherent as reference) to assess the predictors of change in adherence between period 1 and period 2 based on covariates listed above.

#### Sensitivity analyses

The individual health-protective behaviour of carrying a disinfectant was included in the government-mandated health-protective behaviours score and the cross-sectional and longitudinal analyses were repeated for the overall adherence to all four health-protective behaviours.

## Results

Out of a total of 2,992 UK adults recruited into the HEBECO baseline survey, 1,969 participants were included in period 1 analysis, 1,944 at period 2 analysis and 1,622 in the longitudinal analysis. Table 1 shows the unweighted baseline characteristics for the total, included, and excluded samples. There were some differences between included and excluded samples. Included participants were more likely to be older and of white ethnicity. Detailed differences between included and excluded samples for each analysis (period 1, period 2 and longitudinal) are documented in Table 1.

**Table 1:** Sample characteristics

	**Period 1 (3-month analysis)**			**Period 2 (6-month analysis)**		**Longitudinal analysis**	
	Total sample *N* = 2992	Included *N* = 1969	Excluded *N* = 1023	Included *N* = 1944	Excluded *N* = 1048	Included *N* = 1622	Excluded *N* = 1370
Age in years M(SD)	47.24 (15.46)	50.33(14.74)	43.29(15.76)**	51.24(14.39)	41.78(15.50)**	51.57(14.23)	43.61(15.75)**
Female sex, % (N)	68.6 (2054)	69.9 (1376)	66.3 (678)	70.8 (1377)	64.6 (677)**	70.4 (1142)	66.6 (912)
White ethnicity, % (N)	93.7 (2804)	94.9 (1868)	91.5 (936)**	95.5 (1856)	90.5 (948)**	95.5 (1549)	91.6 (1255)**
Living in England, % (N)	85.6 (2562)	85.9 (1691)	85.1 (871)	85.8 (1668)	85.3 (894)	86.1 (1396)	85.1 (1166)
Post-16 qualifications, % (N)	86.7 (2595)	88.0 (1733)	84.3 (862)*	87.4 (1699)	85.5 (896)	88.3 (1433)	84.8 (1162)*
Key worker, % (N)	24.5 (733)	23.7 (466)	26.1 (267)	23.7 (460)	26.0 (273)	23.3 (378)	25.9 (355)
Health problems, % (N)	41.1 (1208)	42.7 (832)	37.8 (376)*	42.1 (809)	39.1 (399)	42.3 (680)	39.6 (528)
Living alone, % (N)	16.8 (504)	17.0 (335)	16.5 (169)	18.1 (352)	17.5 (18)	18.2 (295)	17.6 (75)
Living with people vulnerable to COVID-19, % (N)	18.0 (448)	18.7 (306)	16.6 (142)	13.4 (214)	16.5 (14)	13.9 (184)	12.6 (44)
Income, % (N)							
≥ £50,000	34.4 (714)	34.7 (684)	28.6 (30)	35.5 (690)	29.7 (30)	35.8 (580)	33.1 (140)
< £50,000	57.4 (1191)	57.1 (1125)	62.9 (66)	55.6 (1080)	59.4 (60)	55.5 (901)	56.5 (239)
Prefer not to say	8.1 (169)	8.1 (160)	8.6 (9)	9.0 (174)	10.9 (11)	8.7 (141)	10.4 (44)
Current tobacco smoker, % (N)	13.6 (279)	13.7 (270)	10.2 (9)	13.7 (266)	23.4 (18)*	12.6 (205)	19.8 (79)**
BMI overweight/ obese, % (N)	53.6 (1002)	53.1 (953)	64.5 (49)	53.4 (949)	59.4 (41)	53.0 (793)	56.3 (197)
Quality of life, M(SD)	3.62 (0.78)	3.64 (0.77)	3.60 (0.79)	3.45 (0.78)	3.46 (0.79)	3.45 (0.77)	3.46 (0.79)
Perceived high risk of COVID-19, % (N)	21.1 (438)	20.2 (398)	38.1 (40)**	54.8 (34)	45.2 (28)**	25.3 (411)	27.1 (104)
Diagnosed/suspected COVID-19, % (N)	16.5 (371)	18.0 (354)	24.3 (18)	18.3 (355)	27.4 (17)	17.5 (284)	22.9 (88)*
Experience of social distancing, M(SD)	50.98(22.12)	50.86(22.04)	53.42(23.65)	56.11(24.49)	61.93(25.70)	56.33(24.39)	56.04(25.20)

Significant difference between included and excluded sample adjusted for False Discovery Rate

*M* = Mean, *SD* = Standard deviation

^*^p < 0.05

^**^p < 0.01

### RQ1. Rates of adherence

Detailed descriptive statistics for the overall adherence to government-mandated health- protective behaviours as well as adherence to the four individual health-protective behaviours for period 1 and period 2 are included in Table 2.

**Table 2:** Overall adherence to government-mandated health-protective behaviours and to the four individual health-protective behaviours for period 1 (3-month follow-up) and period 2 (6-month follow-up), weighted and unweighted samples

	**Period 1 Weighted sample *N* = 1863** **% [95%CI]**	**Period 1 Unweighted sample *N* = 1969** **% [95%CI]**	**Period 2 Weighted sample *N* = 1780** **% [95%CI]**	**Period 2 Unweighted sample *N* = 1944** **% [95%CI]**
**Overall adherence to government-mandated health-protective behaviours**				
Overall adherence (0)	12.0 [10.4–13.6]	9.0 [7.7–10.4]	5.1 [4.1–6.1]	2.8 [2.1–3.6]
Overall adherence (1)	18.4 [16.7–20.2]	15.6 [13.4–17.2]	16.9 [15.2–18.7]	15.8 [14.2–17.5]
Overall adherence (2)	26.5 [24.5–28.5]	27.9 [25.9–29.9]	27.7 [25.6–29.7]	27.8 [25.8–29.8]
Overall adherence (3)	45.3 [43.0–47.5]	48.9 [46.7–51.1]	50.3 [48.0–52.7]	53.5 [51.3–55.7]
**Adherence to individual health-protective behaviours**				
Regularly washing hands (always)	63.0 [60.9–65.2]	66.2 [64.1–68.3]	63.8 [61.6–66.0]	66.7 [64.6–68.8]
Wearing masks indoors (always)	80.2 [78.4–82.0]	82.8 [81.1–84.5]	92.4 [91.1–93.6]	95.1 [94.1–96.0]
Maintaining the recommended physical distance (always)	64.0 [61.9–66.2]	69.1 [67.0-]	67.1 [64.9–69.2]	70.3 [68.2–72.3]
Carrying own disinfectant(always)	48.4 [46.2–50.7]	55.5 [53.3–57.7]	50.7 [48.4–53.0]	55.2 [53.0–57.5]

*CI* Confidence intervals, government-mandated health-protective behaviours are regularly washing hands, wearing masks indoors, maintaining the recommended physical distance from others

Overall adherence to government-mandated health-protective behaviours was 45.3% (95%CI 43.0%-47.5%) for period 1 and 50.3% (95%CI 48.0–52.7%) for period 2 (Wilcoxon ranked test Z = -6.335 *p* < 0.001). Rates of adherence to individual health-protective behaviours were also higher at period 2, but there was no perfect adherence (100%) for any of the behaviours assessed. For both period 1 and 2, greatest adherence was found for wearing a mask indoors (80.2%, 95%CI 78.4%-82.0% and 92.4%, 95%CI 91.1%-93.6% respectively), followed by adherence to maintaining the recommended physical distance (64.0%, 95%CI 61.9%-66.2% and 67.1%, 95%CI 64.9%-69.2% respectively) and for regularly washing hands (63.0%, 95%CI 60.9%-65.2% and 63.8%, 95%CI 61.6%-66.0% respectively). Lowest adherence rates for both time periods were reported for carrying own disinfectant (48.4%, 95%CI 46.2%-50.7% and 50.7%, 95%CI 48.4%-53.0% respectively) (Table 2). However, adherence only increased significantly from period 1 to 2 for wearing masks indoors (Z = -13.19, *p* < 0.001) and not for other health-protective behaviours.

### RQ2: Predictors of adherence across period 1 and 2

Greater overall adherence to government-mandated health-protective behaviours was associated with being female, older age, residing in England, having a higher income, living with vulnerable people, and greater perceived high risk of COVID-19 (Table 3). The sensitivity analysis including all four behaviours to assess overall adherence confirmed these results (but country of residence was no longer significant, see Supplementary Table 1).

**Table 3:** Adherence to government-mandated health-protective behaviours at period 1 and period 2. Full GEE model containing all predictor variables adjusted for time, *N*=1622

	Adherence to government-mandated health-protective behaviours QIC = 1517.22	
**All predictors **	**B [95% CI]**	***p***
Female sex (vs other)	**0.22 [0.12, 0.32]**	** < 0.001**
White ethnicity (vs other)	-0.15 [-0.37, 0.07]	0.167
No post-16 qualification (vs yes)	0.14 [0, 0.29]	0.057
Other country of residence (vs England)	**-0.14 [-0.27, -0.13]**	**0.031**
Not being a key worker (vs yes)	0.03 [-0.09, 0.13]	0.660
Living alone (vs not)	-0.07 [-0.22, 0.10]	0.427
Not living with vulnerable people (vs yes)	**-0.13 [-0.24,—0.02]**	**0.019**
Income < £50,000 (vs ≥ £50,000)	**-0.14 [-0.23, -0.04]**	**0.005**
Income ‘Prefer not to say’ (vs ≥ £50,000)	-0.11 [-0.29, 0.06]	0.210
Non-smoker (vs smoker)	0.04 [-0.12, 0.19]	0.648
Obese/overweight (vs other)	0.01 [-0.08, 0.10]	0.760
No health problems (vs yes)	-0.04 [-0.14, 0.06]	0.448
Not perceived high risk of COVID-19 (vs yes)	**-0.23 [-0.32, -0.13]**	**< 0.001**
Not diagnosed/suspected COVID-19 (vs yes)	0.05 [-0.06, 0.16]	0.389
Age (cont.)	**0.09 [0.05, 0.12]**	**< 0.001**
Quality of life	0.05 [-0.09, -0.001]	0.055
Experience of social distancing	0.0002 [-0.0001, 0.001]	0.575

Models also included Time as a covariate. No significant time*predictor interaction improved the model, QIC is a relative (lower is better) measure of goodness of fit, Bold indicates statistical significance. *Β* Beta parameter, *CI* Confidence interval, government-mandated health-protective behaviours are regularly washing hands, wearing masks indoors, maintaining the recommended physical distance from others

Regarding individual health-protective behaviours, greater adherence to regularly washing hands was associated with being female, older age, having no post-16 qualification, higher income, and perceived high risk of COVID-19, while greater adherence to wearing masks indoors was associated only with residing in England. Greater adherence to maintaining the recommended physical distance was associated with being female, older age, having higher income, living with vulnerable people, poorer quality of life and perceived high risk of COVID-19, while greater adherence to carrying own disinfectant was associated with being female, a key worker, and perceived high risk of COVID-19 (Table 4).

**Table 4:** Adherence to four individual health-protective behaviours at period 1 and period 2. Full GEE model containing all predictor variables adjusted for time, *N* = 1622

	**Adherence to washing regularly hands QIC = 449.58**		**Adherence to wearing mask indoors QIC = 281.71**		**Adherence to maintaining the recommended physical distance QIC = 483.139**		**Adherence to carrying own disinfectant QIC = 460.13**	
	**B [95% CI]**	***p***	**B [95% CI]**	***P***	**B [95% CI]**	***p***	**B [95% CI]**	***p***
Female sex (vs other)	**0.15 [0.09, -0.20]**	**< 0.001**	0.04 [-0.01, 0.08]	0.095	**0.07 [0.02, 0.12]**	0.005	**0.29 [0.24, -0.34]**	** < 0.001**
White ethnicity (vs other)	-0.06 [-0.18, 0.06]	0.302	-0.10 [-0.17, -0.02]	0.051	-0.03 [-0.14, 0.09]	0.656	-0.10 [-0.21, 0.01]	0.068
No post-16 qualification (vs yes)	**0.10 [0.02, -0.17]**	0.009	-0.03 [-0.09, 0.04]	0.397	0.04 [-0.03, 0.11]	0.297	0.05 [-0.03, 0.12]	0.248
Other country of residence (vs England)	-0.01 [-0.08, 0.06]	0.832	**-0.19 [-0.26,—0.12]**	**< 0.001**	-0.02 [-0.09, 0.04]	0.463	-0.01 [-0.08, 0.06]	0.739
Not being a key worker (vs yes)	0.01 [-0.05, 0.07]	0.784	0.004 [-0.05, 0.05]	0.988	0.03 [-0.03–0.08]	0.328	**0.07 [0.02, 0.13]**	**0.013**
Living alone (vs not)	0.01 [-0.07, 0.08]	0.890	-0.0001[-0.0004, 0.0002]	0.402	0.03 [-0.04, 0.10]	0.380	-0.04 [-0.12, 0.04]	0.323
Not living with vulnerable people (vs yes)	-0.06 [-0.11, 0.003]	0.063	-0.00001[-0.00003, 0.00001]	0.390	**-0.08 [-0.14–0.03]**	**0.004**	-0.05 [-0.11, 0.01]	0.095
Income < £50,000 (vs ≥ £50,000)	**-0.07 [-0.12, -0.01]**	**0.013**	0.00002[-0.00003, 0.0001]	0.491	**-0.07 [-0.12, -0.02]**	**0.006**	0.01 [-0.04, 0.06]	0.788
Income ‘Prefer not to say’ (vs ≥ £50,000)	-0.04 [-0.13, 0.05]	0.410	0.00001 [-0.0001, 0.0001]	0.731	-0.05 [-0.13, 0.03]	0.235	0.02 [-0.07, 0.12]	0.629
Non-smoker (vs smoker)	-0.001 [-0.07, 0.07]	0.983	0.00003 [-0.0001, 0.0002]	0.618	0.02 [-0.05, 0.09]	0.655	-0.003 [-0.08, 0.07]	0.927
Obese/overweight (vs other)	0.01 [-0.04, 0.06]	0.665	-0.00004 [-0.0001, 0.0001]	0.453	0.01 [-0.04, 0.05]	0.756	0.03 [-0.02, 0.08]	0.201
No health problems (vs yes)	-0.03 [-0.08, 0.03]	0.320	0.02 [-0.03, 0.06]	0.553	-0.03 [-0.08, 0.02]	0.203	0.02 [-0.03, 0.07]	0.501
Not perceived high risk of COVID-19 (vs yes)	**-0.07 [-0.13, -0.02]**	0.006	-0.00001 [-0.00004, 0.00002]	0.312	**-0.14 [-0.19, -0.10]**	**< 0.001**	**-0.15 [-0.20,—0.09]**	**< 0.001**
Not diagnosed/ suspected COVID-19 (vs yes)	0.02 [-0.04, 0.08]	0.535	0.00003 [-0.00003, 0.0001]	0.270	0.01 [-0.05–0.06]	0.821	0.01 [-0.05, 0.07]	0.796
Age	**0.02 [0.003, 0.04]**	**0.021**	0.01 [-0.01, 0.02]	0.323	**0.05 [0.04–0.07]**	**< 0.001**	0.01 [-0.01, 0.03]	0.194
Quality of life	0.001 [-0.03, 0.03]	0.952	-0.0001 [-0.00001, 0.00001]	0.094	**-0.03 [-0.06, -0.01]**	0.006	0.01 [-0.01, 0.04]	0.359
Experience of social distancing	-0.0001 [-0.001, 0.0004]	0.667	-0.0000001 [-0.00001, 0.00001-]	0.789	0.0001 [-0.0002, 0.0004]	0.460	-0.0001 [-0.0004, 0.0002]	0.532

Models also included Time as a covariate. No significant time*predictor interaction improved the model, *QIC* is a relative (lower is better) measure of goodness of fit, Bold indicates statistical significance. *Β* Beta parameter, *CI* Confidence interval. Social distancing 1319 participants, 1888 cases included (1356 excluded = 3244)

### RQ3: Changes in adherence between period 1 and period 2 and factors associated with changes

Among the 1,622 participants present at both time periods, 25.0% (95%CI 22.9–27.1) decreased their adherence to the government-mandated health-protective behaviours between period 1 and 2, 15.7% (95%CI 13.9%-17.4%) increased their adherence, 39.3% (95% CI 36.9–41.7%) always adhered and 20.1% (95% CI 18.2%-22.1%) never adhered.

Relative to never adhering, decreased adherence to government-mandated health-protective behaviours between period 1 and period 2 was associated with residing in England and being obese/overweight. Increased adherence (compared with never adhering) was also associated with being obese/overweight and additionally with higher quality of life score, while always vs never adhering to government-mandated health-protective behaviours at both time periods was associated with being female, older age, and more positive perceived experiences of social distancing (Table 5).

**Table 5:** Correlates of decreased, increased adherence and always adhering to government-mandated health-protective behaviours (regularly washing hands, wearing masks indoors, maintaining the recommended physical distance from others) in relation to never adhering, *N* = 1622

	**Decrease adherence**		**Increase adherence**		**Always adhering**	
	**% [95% CI)**	**aOR [95% CI]**	**% [95% CI)**	**aOR [95% CI]**	**% [95% CI)**	**aOR [95% CI]**
Sex: Other	25.42 [21.51–29.33]	1 (ref.)	16.88 [13.51–20.24]	1 (ref.)	32.92 [28.70–37.14]	1 (ref.)
Female	24.78 [22.27–27.29]	1.18 [0.76–1.84]	15.15 [13.07–17.23]	1.51 [1.01–2.26]	41.94 [39.08–44.81]	2.07*** [0.44–3.03]
Ethnicity: other	19.18 [9.93–28.43]	1 (ref.)	16.44 [7.73–25.15]	1 (ref.)	42.47 [30.85–54.08]	1 (ref.)
White	25.24 [23.08–27.41]	1.07 [0.34–3.38]	15.62 [13.81–17.43]	0.83 [0.43–1.61]	32.12 [36.69–41.56]	0.70 [0.28–1.77]
Post 16 qualification: yes	24.91 [22.67–27.15]	1 (ref.)	16.40 [14.48–18.32]	1 (ref.)	38.31 [35.79–40.83]	1 (ref.)
No	25.40 [19.13–31.66]	0.73 [0.32–1.66]	10.05 [5.73–14.38]	0.50 [0.24–1.04]	46.56 [39.38–53.74]	1.47 [0.82–2.61]
Country of residence: England	23.14 [20.92–25.35]	1 (ref.)	16.40 [14.46–18.35]	1 (ref.)	40.40 [37.825–42.98]	1 (ref.)
Other	36.28 [29.97–42.60]	0.39** [0.18–0.83]	11.06 [6.94–15.18]	1.45 [0.87–2.43]	32.30 [26.16–38.44]	0.66 [0.39–1.10]
Being a key worker: yes	27.51 [22.99–32.04]	1 (ref.)	15.08 [11.46–18.70]	1 (ref.)	35.98 [31.12–40.84]	1 (ref.)
No	24.20 [21.81–26.58]	1.15 [0.70–1.90]	15.84 [13.80–17.87]	1.13 [0.72–1.78]	40.27 [37.54–43.00]	1.11 [0.73–1.69]
Living alone: no	24.61 [22.30–26.92]	1 (ref.)	16.18 [14.21–18.16]	1 (ref.)	39.67 [37.05–42.29]	1 (ref.)
Yes	26.69 [21.49–31.89]	0.97 [0.52–1.81]	13.17 [9.19–17.15]	1.04 [0.60–1.79]	37.37 [31.68–43.06]	1.05 [0.64–1.74]
Living with vulnerable people: Yes	25.90 [20.44–31.35]	1 (ref.)	9.96 [6.23–13.69]	1 (ref.)	48.21 [41.98–54.43]	1 (ref.)
No	24.80 [22.51–27.09]	1.44 [0.74–2.82]	16.70 [14.73–18.68]	0.83 [0.48–1.43]	37.64 [35.07–40.20]	0.70 [0.43–1.14]
Income: ≥ £50,000	22.87 [19.35–26.39]	1 (ref.)	19.42 [16.11–22.73]	1 (ref.)	37.93 [33.87–42.00]	1 (ref.)
< £50,000	25.21 [22.43–27.99]	0.65 [0.41–1.03]	14.04 [11.82–16.27]	0.97 [0.62–1.50]	39.47 [36.34–42.60]	0.87 [0.59–1.30
Prefer not to say	32.06 [23.96–40.16	0.46 [0.16–1.33]	11.45 [5.93–16.98]	2.11 [0.97–4.57]	43.51 [34.91–52.11]	1.28 [0.61–2.69]
Smoking status: smoker	28.57 [22.30–34.84]	1 (ref.)	11.82 [7.34–16.30]	1 (ref.)	36.45 [29.78–43.13]	1 (ref.)
Non-smoker	24.45 [22.21–26.69]	0.97 [0.48–1.98]	16.21 [14.29–18.13]	0.81 [0.44–1.49]	39.68 [37.13–42.22]	1.19 [0.66–2.17]
BMI: other	21.72 [18.63–24.81]	1 (ref.)	16.33 [13.55–19.10]	1 (ref.)	39.21 [35.55–42.88]	1 (ref.)
Obese/ Overweight	26.71 [23.65–29.77]	1.69* [1.09–2.63]	15.53 [13.02–18.04]	1.68** [1.13–2.49]	39.88 [36.49–43.27]	1.31 [0.92–1.87]
Health problems: Yes	24.56 [21.32–27.80]	1 (ref.)	13.09 [10.55–15.63]	1 (ref.)	44.85 [41.11–48.60]	1 (ref.)
No	25.03 [22.23–27.82]	0.96 [0.60–1.54]	17.48 [15.03–19.92]	0.86 [0.57–1.30]	35.17 [32.09–38.25]	0.69 [0.48–1.01]
Perceived high risk of COVID-19: yes	21.10 [16.66–25.55]	1 (ref.)	12.23 [8.66–15.80]	1 (ref.)	51.99 [46.54–57.43]	1 (ref.)
No	25.95 [23.56–28.34]	0.77 [0.41–1.44]	16.53 [14.50–18.55]	1.04 [0.59–1.84]	36.06 [33.44–38.68]	0.61 [0.37–1.01]
Diagnosed/ suspected COVID-19: yes	28.26 [22.92–33.61]	1 (ref.)	14.13 [10.00–18.27]	1 (ref.)	34.42 [28.78–40.06]	1 (ref.)
No	24.29 [22.00–26.59]	1.23 [0.69–2.17]	15.97 [14.01–17.93]	0.94 [0.57–1.54]	40.27 [37.64–42.89]	1.16 [0.73–1.85]
	**M (SD)**	**aOR [95% CI]**	**M (SD)**	**aOR [95% CI]**	**M (SD)**	**aOR [95% CI]**
Age (cont.)	50.57 (14.37)	0.91 [0.78–1.07]	48.70 (15.26)	1.01 [0.86–1.16]	54.44 (12.60)	1.16* [1.02–1.33]
Quality of life	3.77 (0.73)	1.03 [0.79–1.35]	3.61 (0.84)	1.48** [1.16–1.90]	3.58 (0.76)	0.99 [1.06–1.25]
Experience of social distancing	48.94 (21.6)	0.95 [0.86–1.05]	46.54 (21.74)	1.06 [0.97–1.16]	54.86 (21.81)	1.15** [1.06–1.25]

Reference category: never adhering, age presented in decades, quality of life presented in tens

*CI* Confidence interval, *aOR* adjusted odds ratio, *M* Mean, *SD* Standard deviation

^*^p < 0.05

^**^p < 0.01

^***^p < 0.001

Sensitivity analysis of changes in overall adherence to all four health-protective behaviours showed that of the 1,622 participants, 29.6% (95%CI 27.4–31.8) decreased their adherence between period 1 and 2, 21.1% (95%CI 19.1%-23.1%) increased their adherence, 25.1% (95% CI 23.0–27.2%) always adhered and 24.2% (95% CI 22.1%-26.3%) never adhered. Relative to never adhering, decreased adherence to all four health-protective behaviours between period 1 and period 2 was associated with higher income and younger age, while increased adherence (compared with never adhering) was associated with being female, not residing in England, and being obese/overweight. Always vs never adhering was associated with being female, obese/overweight and perceived high risk of COVID-19 (Supplementary Table 3).

**Fig. 2:**
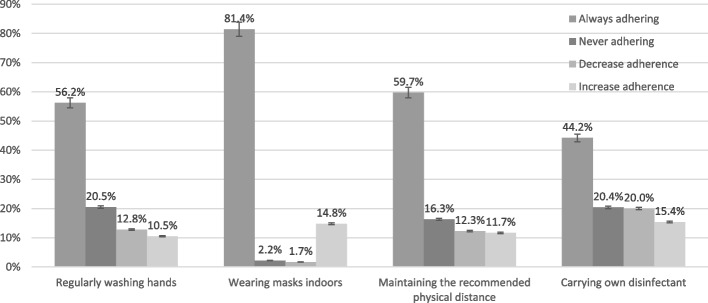
Changes in adherence to each of the four health-protective behaviours between period 1 and period 2, *N* = 1622

Changes in adherence to the individual health-protected behaviours are presented in Fig. 2. Among the 1,622 participants, most participants always adhered to wearing masks indoors, while over half always adhered to regularly washing hands and maintaining the recommended physical distance, and just less than half always adhered to carrying own disinfectant. Similar proportion of participants (~ 20%) never adhered to regularly washing hands and carrying own disinfectant, and a smaller proportion never adhered to maintaining the recommended physical distance (16.3%), while a very small proportion of participants (2.2%) never adhered to wearing masks indoors. Decreased adherence to regularly washing hands and maintaining the recommended physical distance between period 1 and period 2 was observed in 12% of participants, while one in five decreased their adherence to carrying own disinfectant, and a very small proportion of participant (1.7%) decreased adherence to wearing mask indoors between period 1 and 2. Proportions of increased adherence to regularly washing hands and maintaining the recommended physical distance between period 1 and 2 were similar (~ 10%), while similar proportions of participants increased adherence to wearing mask indoors (14.8%) and carrying own disinfectant (15.4%) between period 1 and 2.

Decreased adherence (relative to never adhering) between period 1 and period 2 to carrying own disinfectant was associated with higher income (Supplementary Table 4). Increased adherence (relative to never adhering) between period 1 and period 2 was associated with being female for regularly washing hands and maintaining the recommended physical distance, with not residing in England for wearing masks indoors and maintaining the recommended physical distance, and with perceived high risk of COVID-19 for maintaining the recommended physical distance (Supplementary Table 5).

Relative to never adhering, always adhering for period 1 and period 2 was associated with being female and perceived high risk of COVID-19 for regularly washing hands, maintaining the recommended physical distance, and carrying own disinfectant, with having no post-16 qualifications for regularly washing hands, with more positive perceived experience of social distancing for regularly washing hands and maintaining the recommended physical distance, with older age for maintaining the recommended physical distance, and with being obese/overweight for carrying own disinfectant (Supplementary Table 6).

## Discussion

### Adherence to health-protective behaviours

The current study investigated rates of adherence to health-protective behaviours against COVID-19 in a large convenience sample of UK adults. Data were collected over two periods during the first year of COVID-19 pandemic; at period 1 the first UK lockdown was eased, and less stingiest measures were in place, while at period 2 the second UK lockdown was in place. Our results suggest that there was a significant difference in overall adherence to government-mandated health-protective behaviours between the two time periods, with over half of participants always adhering to government-mandated health-protective behaviours at period 2, but less than half at period 1. Additionally, the analysis regarding changes in adherence between the two time periods showed that almost 40% always adhered, one fifth never adhered, a quarter decreased adherence and around 15% increased adherence between the two time periods.

Examining adherence to the four individual health-protective behaviours, we found that adherence to wearing masks indoors was significant higher at period 2 than period 1, and it was higher at both time points (between 82–95%) compared with the other three health-protective behaviours (regularly washing hands, maintaining the recommended physical distance, and carrying own disinfectant), to which adherence ranged between 55–70%. Lowest adherence was observed for carrying own disinfectant, the only behaviour that was not promoted by the UK government.

The results confirm previous research examining adherence during the first UK lockdown and the period after lockdown, suggesting that people adhere to health-protective behaviours when strictest rules are in place [15]. It also highlights that adherence is higher when clear messages and rules are in place. Thus, it is important to reinforce messaging on adherence when measures are eased to avoid perceptions that remaining measures are somehow unnecessary. This is also evident from the significant difference in adherence to wearing masks between the two time periods as there were mixed messages regarding the protective nature of wearing mask against COVID-19 in the earlier stages of the pandemic [29].

### Factors associated with adherence to health-protective behaviours

With regards to demographic characteristics and in line with previous research, we found that females were more likely than males to adhere to health-protective behaviours [30, 31], which could provide further explanation why males are at increased risk of contracting COVID-19 than women apart from differences in biology [32]. Women have also been shown to be significantly more likely to perceive COVID-19 as a very serious health problem and agree that various health-protective measures are important [30]. Indeed, our findings suggest that individuals who perceived greater risk of COVID-19 showed higher adherence to health-protective behaviours, over and above other characteristics.

Age was also a factor associated with adherence to health-protective behaviours, with younger individuals being less adherent than older ones, as they probably consider themselves fit and strong and less vulnerable to COVID-19. Research has also shown that adherence among younger individuals to health-protective behaviours decreases as they lack clear information and their trust to the regulatory authorities slips [33].

We found that having higher income was a significant factor for always adhering to government-mandated health-protective behaviours as well as to the individual health-protective behaviours of washing hands and maintaining the recommended physical distance. Such findings are in line with speculations that less privileged individuals may be less able to adhere because of work commitments and living in more crowded accommodations. It has also been suggested that those individuals are not convinced of the seriousness of COVID-19 and questioning the effectiveness of COVID-19 guidance [34]. Additionally, individuals living in England were more likely to always adhere to government-mandated health-protective behaviours which could be driven by different regulation in each country during the data collection, while participants living with vulnerable individuals were also more likely to adhere to health-protective behaviours, presumably to shield them from COVID-19.

Taken all these together, it is evident that to achieve high levels of adherence messages need to be targeted to different demographic groups, need to be clear and evolve across the pandemic.

### Strengths and limitations

The study benefited from a large sample size, as well as from including multiple items to measure adherence to COVID-19 health-protective behaviours, a wide range of covariates, and timely assessment during an ongoing pandemic, increasing robustness and reducing the risk of recall bias and confounding. The longitudinal design also allowed measurement of adherence at two timepoints with different social distancing regulations. Previous studies typically focused on the earlier stages of pandemic, when enforcement was stricter and adherence was higher, on average, across the population (i.e., [8, 9, 15]).

The study also has several limitations, including the use of a convenience sample, who may have participated in the study due to a higher interest in the pandemic than the general population. This interest may also be related to a higher tendency to adhere to health-protective behaviours and government guidelines during the COVID-19 pandemic. Additionally, all data were self-reported and thus susceptible to social desirability bias. However, preliminary data indicate that self-reported physical distancing is associated with real-world behaviour [35]. The data also captured adherence at two times during the first year of COVID-19 pandemic. It is important though to monitor adherence at later stages of the pandemic for the successful management of COVID-19, especially given the continue changing COVID-19 situation. Finally, the measures used to capture adherence to health-protective behaviours were non-validated, developed by the researchers, which may have been interpreted differently by participants.

## Conclusions

In conclusion, our findings suggest that adherence to health-protective behaviours was relatively high for most behaviours, but not universal even during periods of stricter enforcement of measures. Highest adherence was observed for wearing masks indoors and was lowest for carrying one’s own disinfectant. The study identified key correlates of adherence, including being female, older age, having higher income, residing in England, living with vulnerable individuals and perceived high risk of COVID-19. Targeted communications to separate demographic groups, such as men and young people, clear and consistent messaging about the rules in place are likely to further increase adherence.

## Supplementary Information

**Additional file 1:** **Supplementary Table 1.** Overall adherence to all four health-protective behaviours for period 1 (3-month follow-up) and period 2 (6-month follow-up), weighted and unweighted samples. **Supplementary Table 2.** Adherence to all four health-protective behaviours at period 1 and period 2. Full GEE model containing all predictor variables adjusted for time. **Supplementary Table 3.** Correlates of decreased, increased adherence and always adhere to any of the four health-protective behaviours (*N*=1622). **Supplementary Table 4.** Correlates of decreased adherence to regularly washing hands, wearing masks indoors, maintaining the recommended physical distance and carrying own disinfectant in reference to never adhere (*N*=1622). **Supplementary Table 5.** Correlates of increased adherence to regularly washing hands, wearing masks indoors, maintaining the recommended physical distance and carrying own disinfectant in reference to never adhere (*N*=1622). **Supplementary Table 6.** Correlates of always adhering to regularly washing hands, wearing masks indoors, maintaining the recommended physical distance and carrying own disinfectant in reference to never adhering (*N*=1622).

## Data Availability

The dataset used and analysed during the current study are available from the corresponding author (Dimitra Kale) on reasonable request.

## Abbreviations

HEBECO: Health behaviours during the COVID-19 study
RQ: Research question
FU1: 1-Month follow-up
FU2: 3-Month follow-up
FU3: 6-Month follow-up
FU4: 12-Month follow-up
BMI: Body mass index
GEE: Generalized estimating equation
QIC: Quasi-likelihood under Independence Model Criterion
CI: Confidence intervals
