# Supplementary material for: Patterns and predictors of adherence to health-protective measures during COVID-19 pandemic in the UK: cross-sectional and longitudinal findings from the HEBECO study
Source: BMC Public Health. 2022 Dec 14;22:2347. doi: 10.1186/s12889-022-14509-7 (PMC9749621; doi:10.1186/s12889-022-14509-7)
Supplement: Supplementary file 1 — Additional file 1: Supplementary Table 1. Overall adherence to all four health-protective behaviours for period 1 (3-month follow-up) and period 2 (6-month follow-up), weighted and unweighted samples. Supplementary Table 2. Adherence to all four health-protective behaviours at period 1 and period 2. Full GEE model containing all predictor variables adjusted for time. Supplementary Table 3. Correlates of decreased, increased adherence and always adhere to any of the four health-protective behaviours (N=1622). Supplementary Table 4. Correlates of decreased adherence to regularly washing hands, wearing masks indoors, maintaining the recommended physical distance and carrying own disinfectant in reference to never adhere (N=1622). Supplementary Table 5. Correlates of increased adherence to regularly washing hands, wearing masks indoors, maintaining the recommended physical distance and carrying own disinfectant in reference to never adhere (N=1622). Supplementary Table 6. Correlates of always adhering to regularly washing hands, wearing masks indoors, maintaining the recommended physical distance and carrying own disinfectant in reference to never adhering (N=1622). [file 12889_2022_14509_MOESM1_ESM.docx]

Supplementary Table 1. Overall adherence to all four health-protective behaviours for period 1 (3-month follow-up) and period 2 (6-month follow-up), weighted and unweighted samples.

|  | **Period 1**  **Weighted sample**  **N=1863**  **% [95%CI]** | **Period 1**  **Unweighted sample**  **N=1969**  **% [95%CI]** | **Period 2**  **Weighted sample**  **N=1780**  **% [95%CI]** | **Period 2**  **Unweighted sample**  **N=1944**  **% [95%CI]** |
| --- | --- | --- | --- | --- |
| **Supplementary analysis: Overall adherence to four health-protective behaviours** | | | | |
| Overall adherence (0) | 8.5 [7.3-9.8] | 6.4 [5.3-7.4] | 4.9 [3.9-5.9] | 2.6 [1.9-3.3] |
| Overall adherence (1) | 14.5 [12.9-16.1] | 12.0 [10.6-13.4] | 14.0 [12.4-15.6] | 12.5 [11.0-14.0] |
| Overall adherence (2) | 19.7 [17.9-21.5] | 18.3 [16.6-20.0] | 21.5 [19.6-23.4] | 19.9 [18.1-21.6] |
| Overall adherence (3) | 27.3 [25.2-29.3] | 28.4 [26.4-30.4] | 21.5 [19.6-23.4] | 25.3 [23.3-27.2] |
| Overall adherence (4) | 30.0 [27.9-32.1] | 34.9 [32.8-37.1] | 38.1 [35.9-40.4] | 39.8 [37.6-42.0] |

Supplementary Table 2. Adherence to all four health-protective behaviours at period 1 and period 2. Full GEE model containing all predictor variables adjusted for time

|  | **QIC=2543.31** | |
| --- | --- | --- |
| **All predictors (N=1622)** | **B [95% CI]** | ***p*** |
| Female sex (vs other) | **0.51 [0.38, 0.64]** | **<0.001** |
| White ethnicity (vs other) | -0.26 [-0.53, 0.02] | 0.064 |
| No post-16 qualification (vs yes) | 0.09 [-0.003, 0.37] | 0.054 |
| Other country of residence (vs England) | -0.16 [-0.33, 0.01] | 0.069 |
| Not being a key worker (vs yes) | 0.10 [-0.05, 0.24] | 0.191 |
| Living alone (vs not) | -0.11 [-0.31, 0.09] | 0.270 |
| Not living with vulnerable people (vs yes) | **-0.17 [-0.31, -0.02]** | **0.023** |
| Income <£50,000 (vs ≥£50,000) | **-0.13 [-0.25, -0.01]** | **0.039** |
| Income ‘Prefer not to say’ (vs ≥£50,000) | -0.11 [-0.34, 0.13] | 0.372 |
| Non-smoker (vs smoker) | 0.03 [-0.16, 0.22] | 0.765 |
| Obese/overweight (vs other) | 0.04 [-0.08, 0.16] | 0.486 |
| No health problems (vs yes) | -0.02 [-0.15, 0.11] | 0.727 |
| Not perceived high risk of COVID-19 (vs yes) | **-0.37 [-0.49, -0.25]** | **<0.001** |
| Not diagnosed/suspected COVID-19 (vs yes) | 0.06 [-0.09, 0.21] | 0.417 |
| Age (cont.) | **0.10 [0.05, 0.15]** | **<0.001** |
| Quality of life | -0.03 [-0.09, 0.02] | 0.247 |
| Experience of social distancing | 0.0002 [-0.001, 0.001] | 0.276 |

Models also included Time as a covariate. No significant time*predictor interaction improved the model, QIC is a relative (lower is better) measure of goodness of fit, Bold indicates statistical significance. Β: Beta parameter, CI: Confidence Interval.

Supplementary Table 3. Correlates of decreased, increased adherence and always adhere to any of the four health-protective behaviours (N=1622)

|  | **Decrease adherence** | | **Increase adherence** | | **Always adhere** | |
| --- | --- | --- | --- | --- | --- | --- |
|  | **%**  **[95% CI)** | **aOR**  **[95% CI]** | **%**  **[95% CI)** | **aOR**  **[95% CI]** | **%**  **[95% CI)** | **aOR**  **[95% CI]** |
| Sex: Other | 12.71  [10.27-14.07] | 1 (ref.) | 10.63  [7.86-13.39] | 1 (ref.) | 14.79  [11.60-17.98] | 1 (ref.) |
| Female | 12.17  [10.27-14.07] | 1.29  [0.87-1.89] | 12.17  [10.27-14.07] | **1.75****  **[1.21-2.51]** | 29.42  [26.78-32.07] | **4.48*****  **[2.90-6.91]** |
| Ethnicity: other | 13.70  [5.62-21.78] | 1 (ref.) | 12.33  [4.60-20.05] | 1 (ref.) | 31.51  [20.59-42.42] | 1 (ref.) |
| White | 12.27  [10.63-13.90] | 0.68  [0.24-1.97] | 11.68  [10.08-13.29] | 1.01  [0.33-3.03] | 24.79  [22.64-26.94] | 0.46  [0.16-1.29] |
| Post 16 qual.: yes | 12.42  [10.71-14.13] | 1 (ref.) | 12.07  [10.38-13.76] | 1 (ref.) | 24.49  [22.26-26.72] | 1 (ref.) |
| No | 11.64  [7.03-16.25] | 0.90  [0.45-1.79] | 8.99  [4.88-13.11] | 0.93  [0.52-1.66] | 29.63  [23.06-36.20] | 1.45  [0.81-2.59] |
| Country of residence: England | 12.54  [10.80-14.27] | 1 (ref.) | 11.75  [10.06-13.44] | 1 (ref.) | 25.57  [23.28-27.86] | 1 (ref.) |
| Other | 11.06  [6.94-15.18] | 0.63  [0.33-1.17] | 11.50  [7.31-15.70] | **1.65***  **[1.02-2.66]** | 22.12  [16.67-27.58] | 0.80  [0.45-1.41] |
| Being a key worker: yes | 11.38  [8.16-14.59] | 1 (ref.) | 15.34  [11.69-18.99] | 1 (ref.) | 23.28  [19.00-27.56] | 1 (ref.) |
| No | 12.62  [10.77-14.47] | 1.35  [0.86-2.11] | 10.61  [8.90-12.32] | 1.36  [0.89-2.08] | 25.64  [23.21-28.07] | 1.11  [0.71-1.74] |
| Living alone: no | 12.68  [10.89-14.46] | 1 (ref.) | 11.48  [9.78-13.19] | 1 (ref.) | 25.65  [23.31-27.99] | 1 (ref.) |
| Yes | 10.68  [7.04-14.31] | 0.83  [0.48-1.43] | 12.81  [8.88-16.74] | 0.91  [0.56-1.48] | 22.42  [17.51-27.33] | 0.93  [0.55-1.55] |
| Living with vulnerable people: Yes | 8.37  [4.92-11.82] | 1 (ref.) | 9.96  [6.23-13.69] | 1 (ref.) | 33.07  [27.21-38.93] | 1 (ref.) |
| No | 13.06  [11.27-14.84] | 1.39  [0.78-2.51] | 12.04  [10.31-13.76] | 0.77  [0.47-1.27] | 23.63  [21.38-25.88] | 0.64  [0.39-1.07] |
| Income: ≥£50,000 | 14.70  [11.73-17.67] | 1 (ref.) | 11.62  [8.93-14.30] | 1 (ref.) | 22.87  [19.35-26.39] | 1 (ref.) |
| <£50,000 | 11.49  [9.45-13.53] | **0.56****  **[0.37-0.85]** | 11.28  [9.25-13.30] | 1.03  [0.69-1.54] | 25.53  [22.74-28.32] | 1.01  [0.66-1.56] |
| Prefer not to say | 8.40  [3.58-13.21] | 0.75  [0.32-1.79] | 15.27  [9.03-21.51] | **2.33***  **[1.10-4.90]** | 31.30  [23.25-39.34] | 1.78  [0.80-3.93] |
| Smoking status: Smoker | 7.88  [4.14-11.62] | 1 (ref.) | 16.75  [11.57-21.93] | 1 (ref.) | 22.66  [16.85-28.47] | 1 (ref.) |
| Non-smoker | 12.97  [11.22-14.72] | 1.15  [0.61-2.17] | 10.99  [9.36-12.62] | 0.95  [0.54-1.65] | 25.44  [23.17-27.71] | 1.52  [0.80-2.90] |
| BMI: other | 12.83  [10.32-15.34] | 1 (ref.) | 10.50  [8.20-12.79] | 1 (ref.) | 22.74  [19.60-25.88] | 1 (ref.) |
| Obese/overweight | 11.80  [9.57-14.03] | 1.27  [0.86-1.87] | 11.93  [9.68-14.17] | **1.63****  **[1.14-2.33]** | 27.45  [24.36-30.54] | **1.66****  **[1.14-2.43]** |
| Health problems: Yes | 10.00  [7.74-12.26] | 1 (ref.) | 11.47  [9.07-13.87] | 1 (ref.) | 29.41  [25.98-32.85] | 1 (ref.) |
| No | 14.02  [11.78-16.26] | 0.96  [0.64-1.45] | 11.76  [9.68-13.84] | 0.91  [0.62-1.32] | 22.11  [19.44-24.79] | 0.79  [0.53-1.18] |
| Perceived high risk of Covid-19: yes | 7.95  [5.00-10.90] | 1 (ref.) | 5.81  [3.26-8.36] | 1 (ref.) | 36.39  [31.15-41.63] | 1 (ref.) |
| No | 13.44  [11.58-15.30] | 0.83  [0.48-1.44] | 13.20  [11.36-15.05] | 0.95  [0.58-1.56] | 22.24  [19.97-24.51] | **0.56***  **[0.34-0.91]** |
| Diagnosed/suspected Covid-19: yes | 12.68  [8.73-16.63] | 1 (ref.) | 13.77  [9.68-17.86] | 1 (ref.) | 23.91  [18.85-28.98] | 1 (ref.) |
| No | 12.26  [10.50-14.01] | 1.09  [0.64-1.85] | 11.29  [9.60-12.99] | 0.76  [0.47-1.21] | 25.33  [23.01-27.66] | 0.85  [0.51-1.42] |
|  | **M (SD)** | **aOR**  **[95% CI]** | **M (SD)** | **aOR**  **[95% CI]** | **M (SD)** | **aOR**  **[95% CI]** |
| Age | 49.11  (15.77) | **0.85***  **[0.74-0.98]** | 48.92  (13.68) | 0.96  [0.84-1.10] | 54.93  (11.76) | 1.12  [0.96-1.30] |
| Quality of life | 3.58  (0.77) | 1.18  [0.93-1.50] | 3.74  (0.70) | 1.38  [1.10-1.09] | 3.55(0.75) | 0.99  [0.78-1.24] |
| Experience of social distancing | 48.30  (22.33) | 0.94  [0.86-1.03] | 46.84  (20.28) | 1.01  [0.93-1.09] | 54.01  (21.69) | 1.05  [0.97-1.15] |

Reference category: never adhere, age presented in decades, quality of life presented in tens, *p<0.05, **p<0.01, ***p<0.001, CI=confidence interval, aOR=adjusted odds ratio, M=mean, SD=Standard deviation.

Supplementary Table 4. Correlates of decreased adherence to regularly washing hands, wearing masks indoors, maintaining the recommended physical distance and carrying own disinfectant in reference to never adhere (N=1622)

|  | **Regularly washing hands** | | **Wearing masks indoors** | | **Maintaining the recommended physical distance** | | **Carrying own disinfectant** | |
| --- | --- | --- | --- | --- | --- | --- | --- | --- |
|  | **%**  **[95% CI)** | **aOR**  **[95% CI]** | **%**  **[95% CI)** | **aOR**  **[95% CI]** | **%**  **[95% CI)** | **aOR**  **[95% CI]** | **%**  **[95% CI)** | **aOR**  **[95% CI]** |
| Sex: Other | 15.83  [12.56-19.11] | 1 (ref.) | 2.29  [0.95-3.64] | 1 (ref.) | 12.71  [9.72-15.70] | 1 (ref.) | 27.29  [23.29-31.29] | 1 (ref.) |
| Female | 11.56  [9.70-13.42] | 1.14  [0.72-1.78] | 1.40  [0.72-2.08] | 0.60  [0.14-2.66] | 12.17  [10.27-14.07] | 1.70  [1.04-2.79] | 16.99  [14.81-19.17] | 1.21  [0.81-1.79] |
| Ethnicity: other | 20.55  [11.06-30.04] | 1 (ref.) | 2.74  [-1.10-6.57] | 1 (ref.) | 13.70  [5.62-21.78] | 1 (ref.) | 23.29  [13.36-33.22] | 1 (ref.) |
| White | 12.46  [10.81-14.11] | 0.17  [0.04-0.81] | 1.61  [0.99-2.24] | - | 12.27  [10.63-13.90] | 0.35  [0.06-1.86] | 19.88  [17.89-21.87] | 0.46  [0.15-1.39] |
| Post 16 qualification: yes | 13.12  [11.37-14.87] | 1 (ref.) | 1.67  [1.01-2.34] | 1 (ref.) | 12.42  [10.71-14.13] | 1 (ref.) | 20.10  [18.02-22.17] | 1 (ref.) |
| No | 10.58  [6.16-15.01] | 2.08  [0.92-4.74] | 1.59  [-0.21-3.39] | 0.62  [0.05-8.45] | 11.64  [7.03-16.25] | 1.08  [0.46-2.51] | 19.58  [13.87-25.29] | 1.95  [0.98-3.89] |
| Country of residence: England | 13.54  [11.74-15.34] | 1 (ref.) | 1.72  [1.04-2.40] | 1 (ref.) | 12.54  [10.80-14.27] | 1 (ref.) | 20.27  [18.16-22.38] | 1 (ref.) |
| Other | 8.41  [4.76-12.05] | 0.28  [0.28-1.22] | 1.33  [-0.18-2.83] | 1.27  [0.07-4.82] | 11.06  [6.94-15.18] | 0.72  [0.36-1.45] | 18.58  [13.47-23.69] | 1.42  [0.76-2.63] |
| Being a key worker: yes | 11.90  [8.63-15.18] | 1 (ref.) | 1.59  [0.32-2.85] | 1 (ref.) | 11.38  [8.16-14.59] | 1 (ref.) | 20.11  [16.05-24.16] | 1 (ref.) |
| No | 13.10  [11.23-14.98] | 1.37  [0.81-2.31] | 1.69  [0.97-2.40] | 2.19  [0.41-11.71] | 12.62  [10.77-14.47] | 1.14  [0.65-1.99] | 20.02  [17.79-22.24] | 1.30  [0.82-2.07] |
| Living alone: no | 12.90  [11.10-14.70] | 1 (ref.) | 1.64  [0.96-2.32] | 1 (ref.) | 12.68  [10.89-14.46] | 1 (ref.) | 20.66  [18.49-22.83] | 1 (ref.) |
| Yes | 12.46  [8.57-16.34] | 1.01  [0.53-1.90] | 1.78  [0.22-3.33] | 0.46  [0.04-5.98] | 10.68  [7.04-14.31] | 0.91  [0.46-1.80] | 17.08  [12.65-21.51] | 0.58  [0.32-1.05] |
| Living with vulnerable people: Yes | 9.16  [5.57-12.76] | 1 (ref.) | 1.59  [0.03-3.15] | 1 (ref.) | 8.37  [4.92-11.82] | 1 (ref.) | 15.54  [11.03-20.05] | 1 (ref.) |
| No | 13.49  [11.68-15.30] | 0.96  [0.49-1.87] | 1.68  [1.00-2.36] | 0.43  [0.03-5.71] | 13.06  [11.27-14.84] | 1.50  [0.70-3.23] | 20.86  [18.71-23.01] | 1.41  [0.78-2.54] |
| Income: ≥£50,000 | 15.25  [12.23-18.26] | 1 (ref.) | 0.91  [0.11-1.70] | 1 (ref.) | 14.70  [11.73-17.67] | 1 (ref.) | 25.77  [22.11-29.43] | 1 (ref.) |
| <£50,000 | 11.91  [9.84-13.99] | 0.70  [0.43-1.13] | 2.02  [1.12-2.92] | 1.32  [0.22-8.01] | 11.49  [9.45-13.53] | 0.74  [0.44-1.24] | 17.23  [14.82-19.65] | **0.57***  **[0.37-0.88]** |
| Prefer not to say | 9.16  [4.15-14.17] | 0.80  [0.28-2.28] | 2.29  [-0.31-4.89] | 1.55  [0.06-39.26] | 8.40  [3.58-13.21] | 0.59  [0.20-1.77] | 16.03  [9.66-22.40] | 0.78  [0.34-1.82] |
| Smoking status: Smoker | 11.82  [7.34-16.30] | 1 (ref.) | 1.48  [-0.20-3.15] | 1 (ref.) | 7.88  [4.14-11.62] | 1 (ref.) | 18.72  [13.31-24.13] | 1 (ref.) |
| Non-smoker | 12.97  [11.22-14.72] | 1.25  [0.61-2.56] | 1.69  [1.02-2.36] | 2.40  [0.35-16.61] | 12.97  [11.22-14.72] | 1.49  [0.65-3.41] | 20.23  [18.13-22.32] | 1.06  [0.57-1.97] |
| BMI: other | 13.41  [10.85-15.97] | 1 (ref.) | 1.31  [0.46-2.17] | 1 (ref.) | 12.83  [10.32-15.34] | 1 (ref.) | 22.30  [19.18-25.43] | 1 (ref.) |
| Obese/overweight | 12.30  [10.02-14.57] | 1.33  [0.84-2.10] | 2.11  [1.12-3.11] | 1.29  [0.29-5.68] | 11.80  [9.57-14.03] | 1.10  [0.68-1.79] | 18.26  [15.59-20.94] | 1.02  [0.69-1.53] |
| Health problems: Yes | 11.32  [8.94-13.71] | 1 (ref.) | 2.21  [1.10-3.31] | 1 (ref.) | 14.02  [11.78-16.26] | 1 (ref.) | 19.41  [16.43-22.39] | 1 (ref.) |
| No | 14.02  [11.78-16.26] | 1.11  [0.68-1.80] | 1.29  [0.57-2.02] | 0.27  [0.06-1.22] | 10.00  [7.74-12.26] | 0.95  [0.56-1.61] | 20.71  [18.10-23.33] | 1.32  [0.87-2.02] |
| Perceived high risk of Covid-19: yes | 11.01  [7.60-14.42] | 1 (ref.) | 0.31  [-0.30-0.91] | 1 (ref.) | 7.95  [5.00-10.90] | 1 (ref.) | 15.29  [11.37-19.21] | 1 (ref.) |
| No | 13.25  [11.43-15.13] | 0.61  [0.32-1.19] | 2.01  [1.24-2.77] | 6.20  [0.55-69.55] | 13.44  [11.58-15.30] | 0.54  [0.23-1.27] | 21.24  [19.01-23.47] | 0.57  [0.31-1.04] |
| Diagnosed/  suspected Covid-19: yes | 14.13  [10.00-18.27] | 1 (ref.) | 1.45  [0.03-2.87] | 1 (ref.) | 12.68  [8.73-16.63] | 1 (ref.) | 20.65  [15.85-25.46] | 1 (ref.) |
| No | 12.56  [10.78-14.33] | 0.73  [0.40-1.31] | 1.71  [1.02-2.40] | 3.43  [0.31-38.43] | 12.26  [10.50-14.01] | 0.95  [0.50-1.82] | 19.91  [17.77-22.05] | 0.86  [0.51-1.45] |
|  | **M (SD)** | **aOR**  **[95% CI]** | **M (SD)** | **aOR**  **[95% CI]** | **M (SD)** | **aOR**  **[95% CI]** | **M (SD)** | **aOR**  **[95% CI]** |
| Age | 48.07 (16.07) | 0.86  [0.73-1.02] | 47.56 (16.20) | 0.83  [0.48-1.41] | 49.11 (15.77) | 1.09  [0.92-1.30] | 49.60 (14.84) | 0.97  [0.84-1.12] |
| Quality of life | 3.62 (0.85) | 0.95  [0.72-1.26] | 3.34 (0.96) | 1.06  [0.46-2.45] | 3.58 (0.77) | 0.81  [0.60-1.10] | 3.63 (0.82) | 1.06  [0.83-1.35] |
| Experience of social distancing | 46.19 (21.24) | 0.97  [0.87-1.08] | 42.26 (20.93) | 1.00  [0.72-1.39] | 48.30 (22.33) | 1.02  [0.91-1.14] | 47.99 (21.52) | 0.95  [0.86-1.04] |

Reference category: never adhere, age presented in decades, quality of life presented in tens, *p<0.05, **p<0.01, ***p<0.001, CI=confidence interval, aOR=adjusted odds ratio, M=mean, SD=Standard deviation.

Supplementary Table 5. Correlates of increased adherence to regularly washing hands, wearing masks indoors, maintaining the recommended physical distance and carrying own disinfectant in reference to never adhere (N=1622)

|  | **Regularly washing hands** | | **Wearing masks indoors** | | **Maintaining the recommended physical distance** | | **Carrying own disinfectant** | |
| --- | --- | --- | --- | --- | --- | --- | --- | --- |
|  | **%**  **[95% CI)** | **aOR**  **[95% CI]** | **%**  **[95% CI)** | **aOR**  **[95% CI]** | **%**  **[95% CI)** | **aOR**  **[95% CI]** | **%**  **[95% CI)** | **aOR**  **[95% CI]** |
| Sex: Other | 9.38  [6.76-11.99] | 1 (ref.) | 17.08  [13.70-20.46] | 1 (ref.) | 10.63  [7.86-13.39] | 1 (ref.) | 17.92  [14.47-21.36] | 1 (ref.) |
| Female | 10.95  [9.13-12.76] | **2.20****  **[1.29-3.74]** | 13.84  [11.83-15.84] | 0.97  [0.32-2.99] | 12.17  [10.27-14.07] | **1.98***  **[1.17-3.38]** | 14.27  [12.24-16.31] | **2.09****  **[1.34-3.26]** |
| Ethnicity: other | 9.59  [2.67-16.51] | 1 (ref.) | 8.22  [1.77-14.67] | 1 (ref.) | 11.68  [10.08-13.29] | 1 (ref.) | 13.70  [5.62-21.78] | 1 (ref.) |
| White | 10.52  [8.99-12.05] | 0.51  [0.07-3.79] | 15.11  [13.32-16.89] | 17.63  [0.94-33.51] | 12.33  [4.60-20.05] | 0.24  [0.04-1.31] | 15.43  [13.63-17.23] | 0.66  [0.18-2.40] |
| Post 16 qualification: yes | 10.40  [8.82-11.98] | 1 (ref.) | 14.65  [12.82-16.49] | 1 (ref.) | 12.07  [10.38-13.76] | 1 (ref.) | 15.56  [13.68-17.44] | 1 (ref.) |
| No | 11.11  [6.59-15.63] | 0.80  [0.41-1.58] | 15.87  [10.62-21.13] | 1.13  [0.21-6.26] | 8.99  [4.88-13.11] | 0.52  [0.19-1.42] | 13.76  [8.80-18.71] | 1.37  [0.65-2.90] |
| Country of residence: England | 9.81  [8.25-11.38] | 1 (ref.) | 12.18  [10.46-13.90] | 1 (ref.) | 11.75  [10.06-13.44] | 1 (ref.) | 14.54  [12.69-16.39] | 1 (ref.) |
| Other | 14.60  [9.96-19.24] | 1.49  [0.78-2.82] | 30.97  [24.90-37.05] | **8.03***  **[1.01-13.94]** | 11.50  [7.31-15.70] | 0.64  [0.37-1.09] | 20.35  [15.06-25.64] | **2.54****  **[1.39-4.66]** |
| Being a key worker: yes | 9.52  [6.55-12.50] | 1 (ref.) | 16.93  [13.13-20.73] | 1 (ref.) | 15.34  [11.69-18.99] | 1 (ref.) | 14.81  [11.22-18.41] | 1 (ref.) |
| No | 10.77  [9.05-12.50] | 1.57  [0.85-2.88] | 14.15  [12.21-16.09] | 2.03  [0.65-6.30] | 10.61  [8.90-12.32] | 0.70  [0.40-1.23] | 15.51  [13.50-17.53] | 1.17  [0.70-1.96] |
| Living alone: no | 9.99  [8.39-11.60] | 1 (ref.) | 14.09  [12.23-15.96] | 1 (ref.) | 11.48  [9.78-13.19] | 1 (ref.) | 14.84  [12.93-16.74] | 1 (ref.) |
| Yes | 12.81  [8.88-16.74] | 0.76  [0.38-1.52] | 18.15  [13.62-22.68] | 2.91  [0.59-14.31] | 12.81  [8.88-16.74] | 0.72  [0.35-1.51] | 17.79  [13.29-22.29] | 1.14  [0.64-2.02] |
| Living with vulnerable people: Yes | 11.95  [7.91-15.99] | 1 (ref.) | 13.15  [8.94-17.36] | 1 (ref.) | 9.96  [6.23-13.69] | 1 (ref.) | 16.33  [11.73-20.94] | 1 (ref.) |
| No | 10.21  [8.61-11.82] | 0.73  [0.37-1.43] | 15.10  [13.20-17.00] | 0.26  [0.03-2.18] | 12.05  [10.31-13.76] | 0.98  [0.47-2.05] | 15.17  [13.27-17.07] | 0.82  [0.45-1.50] |
| Income: ≥£50,000 | 7.44  [5.24-9.64] | 1 (ref.) | 14.70  [11.73-17.67] | 1 (ref.) | 11.62  [13.30-6.97] | 1 (ref.) | 12.89  [10.08-15.69] | 1 (ref.) |
| <£50,000 | 11.17  [9.15-13.19] | 1.68  [0.93-3.01] | 15.11  [12.81-17.40] | 0.35  [0.10-1.26] | 11.28  [9.25-13.30] | 0.92  [0.52-1.60] | 17.13  [14.71-19.54] | 1.15  [0.70-1.88] |
| Prefer not to say | 18.32  [11.61-25.03] | **5.20****  **[2.03-13.3]** | 12.98  [7.15-18.81] | 0.96  [0.09-10.02] | 15.27  [9.03-21.51] | 1.65  [0.62-4.38] | 12.86  [7.15-18.81] | 1.48  [0.60-3.66] |
| Smoking status: Smoker | 9.85  [13.99-5.39] | 1 (ref.) | 15.27  [10.28-20.26] | 1 (ref.) | 16.75  [11.57-21.93] | 1 (ref.) | 16.75  [11.57-21.93] | 1 (ref.) |
| Non-smoker | 10.57  [8.97-12.17] | 1.45  [0.65-3.25] | 14.73  [12.88-16.57] | 2.62  [0.74-9.25] | 10.99  [9.36-12.62] | 0.66  [0.31-1.39] | 15.15  [13.28-17.02] | 1.20  [0.60-2.37] |
| BMI: other | 9.77  [7.54-11.99 | 1 (ref.) | 12.97  [10.45-15.49] | 1 (ref.) | 10.50 [8.20-12.79] | 1 (ref.) | 15.60  [12.88-18.32] | 1 (ref.) |
| Obese/overweight | 10.68  [8.54-12.82] | 1.02  [0.62-1.67] | 15.90  [13.37-18.43] | 1.10  [0.38-3.18] | 11.93  [9.68-14.17] | 1.13  [0.68-1.89] | 15.16  [12.67-17.64] | 1.06  [0.68-1.64] |
| Health problems: Yes | 10.44  [8.14-12.75] | 1 (ref.) | 15.15  [12.45-17.85] | 1 (ref.) | 11.47  [9.07-13.87] | 1 (ref.) | 16.03  [13.26-18.79] | 1 (ref.) |
| No | 10.46  [8.49-12.44] | 1.12  [0.66-1.90] | 14.46  [12.19-16.72] | 0.72  [0.24-2.18] | 11.76  [9.68-13.84] | 0.64  [0.37-1.09] | 14.89  [12.59-17.18] | 1.18  [0.75-1.87] |
| Perceived high risk of Covid-19: yes | 9.79  [6.55-13.02] | 1 (ref.) | 13.76  [10.01-17.51] | 1 (ref.) | 5.81  [3.26-8.36] | 1 (ref.) | 16.51  [12.47-20.56] | 1 (ref.) |
| No | 10.66  [8.97-12.34] | 0.91  [0.44-1.90] | 15.06  [13.11-17.01] | 1.39  [0.37-5.27] | 13.20  [11.36-15.05] | 1.44  [0.52-3.99] | 15.06  [13.11-17.01] | **0.53***  **[0.28-0.99]** |
| Diagnosed/  suspected Covid-19: yes | 11.96  [8.10-15.81] | 1 (ref.) | 20.29  [15.52-25.06] | 1 (ref.) | 13.77  [17.86-9.74] | 1 (ref.) | 15.94  [11.60-20.29] | 1 (ref.) |
| No | 10.18  [8.56-11.80] | 0.61  [0.32-1.14] | 13.67  [11.83-15.51] | 0.64  [0.17-2.48] | 11.29  [9.60-12.99] | 0.79  [0.41-1.50] | 15.23  [13.31-17.15] | 0.82  [0.46-1.46] |
|  | **M (SD)** | **aOR**  **[95% CI]** | **M (SD)** | **aOR**  **[95% CI]** | **M (SD)** | **aOR**  **[95% CI]** | **M (SD)** | **aOR**  **[95% CI]** |
| Age | 52.22 (14.70) | 1.02  [0.85-1.22] | 51.42 (14.01) | 0.80  [0.53-1.21] | 48.92 (13.68) | 1.18  [0.98-1.41] | 51.64 (14.64) | 1.07  [0.91-1.27] |
| Quality of life | 3.75 (0.72) | 1.39  [1.02-1.91] | 3.73 (0.77) | 2.02  [1.08-3.76] | 3.74 (0.70) | 1.10  [0.80-1.53] | 3.63 (0.80) | 1.08  [0.82-1.41] |
| Experience of social distancing | 50.52 (21.98) | 1.06  [0.95-1.97] | 47.82 (22.20) | 1.15  [0.91-1.45] | 46.84 (20.28) | 1.09  [0.97-1.23] | 50.44 (21.67) | 0.98  [0.89-1.08] |

Reference category: never adhere, age presented in decades, quality of life presented in tens, *p<0.05, **p<0.01, ***p<0.001, CI=confidence interval, aOR=adjusted odds ratio, M=mean, SD=Standard deviation.

Supplementary Table 6. Correlates of always adhering to regularly washing hands, wearing masks indoors, maintaining the recommended physical distance and carrying own disinfectant in reference to never adhering (N=1622)

|  | **Regularly washing hands** | | **Wearing masks indoors** | | **Maintaining the recommended physical distance** | | **Carrying own disinfectant** | |
| --- | --- | --- | --- | --- | --- | --- | --- | --- |
|  | **%**  **[95% CI)** | **aOR**  **[95% CI]** | **%**  **[95% CI)** | **aOR**  **[95% CI]** | **%**  **[95% CI)** | **aOR**  **[95% CI]** | **%**  **[95% CI)** | **aOR**  **[95% CI]** |
| Sex: Other | 46.67  [42.19-51.15] | 1 (ref.) | 78.54  [74.86-82.23] | 1 (ref.) | 55.21  [50.74-59.67] | 1 (ref.) | 23.13  [19.34-26.91] | 1 (ref.) |
| Female | 60.16  [57.31-63.00] | **2.35*****  **[1.67-3.31]** | 82.57  [80.37-84.78] | 1.04  [0.36-3.06] | 61.56  [58.73-64.38] | **2.29 *****  **[1.56-3.36]** | 53.06  [50.17-55.96] | **5.67*****  **[3.85-8.36]** |
| Ethnicity: other | 58.90  [47.35-70.46] | 1 (ref.) | 84.93  [76.53-93.34] | 1 (ref.) | 61.64  [50.22-73.07] | 1 (ref.) | 52.05  [40.32-63.79] | 1 (ref.) |
| White | 56.04  [53.56-58.51] | 0.23  [0.05-0.99] | 81.21  [79.27-83.16] | 2.45  [0.28-21.65] | 59.59  [57.14-62.03] | 0.30  [0.07-1.36] | 43.83  [41.36-46.31] | 0.61  [0.21-1.78] |
| Post 16 qualification: yes | 55.27  [52.69-57.85] | 1 (ref.) | 81.58  [79.57-83.59] | 1 (ref.) | 59.04  [56.49-61.59] | 1 (ref.) | 44.03  [41.46-46.61] | 1 (ref.) |
| No | 62.96  [56.02-69.91] | **2.25***  **[1.20-4.20]** | 79.89  [74.13-85.66] | 1.72  [0.35-8.54] | 64.55  [57.67-71.43] | 1.35  [0.71-2.54] | 45.50  [38.34-52.67] | 1.73  [0.93-3.22] |
| Country of residence: England | 56.30  [53.70-58.91] | 1 (ref.) | 83.88  [81.95-85.81] | 1 (ref.) | 59.74  [57.17-62.32] | 1 (ref.) | 44.20  [41.59-46.81] | 1 (ref.) |
| Other | 55.31  [48.78-61.84] | 0.92  [0.57-1.47] | 65.93  [59.70-72.16] | 2.26  [0.29-17.61] | 59.29  [52.84-65.75] | 0.71  [0.43-1.20] | 44.25  [37.72-50.77] | 1.40  [0.80-2.44] |
| Being a key worker: yes | 56.08  [51.06-61.11] | 1 (ref.) | 79.63  [75.55-83.71] | 1 (ref.) | 55.29  50.26-60.33] | 1 (ref.) | 42.86  [37.85-47.87] |  |
| No | 56.19  [53.43-58.95] | 1.20  [0.82-1.75] | 81.91  [79.77-84.06] | 1.68  [0.58-4.87] | 61.01  [58.30-63.73] | 1.00  [0.64-1.56] | 44.61  [41.85-47.38] | 1.24  [0.82-1.88] |
| Living alone: no | 57.05  [54.39-59.70] | 1 (ref.) | 82.10  [80.05-84.16] | 1 (ref.) | 59.43  [56.80-62.06] | 1 (ref.) | 44.44  [41.78-47.11] |  |
| Yes | 51.96  [46.08-57.83] | 1.00  [0.63-1.58] | 77.94  [73.06-82.81] | 1.99  [0.43-9.28] | 60.85  [55.11-66.60] | 1.03  [0.61-1.74] | 43.06  [37.24-48.89] | 0.95  [0.59-1.56] |
| Living with vulnerable people: Yes | 62.15  [56.11-68.19] | 1 (ref.) | 84.46  [79.95-88.97] | 1 (ref.) | 70.52  [64.84-76.20] | 1 (ref.) | 51.39  [45.17-57.62] | 1 (ref.) |
| No | 55.07  [52.43-57.71] | 0.71  [0.44-1.14] | 80.82  [78.73-82.90] | 0.20  [0.03-2.05] | 57.70  [55.08-60.31] | 0.67  [0.39-1.15] | 42.89  [40.27-45.51] | 0.76  [0.46-1.25] |
| Income: ≥£50,000 | 55.35  [51.19-59.52] | 1 (ref.) | 83.48  [80.37-86.59] | 1 (ref.) | 57.89  [53.76-62.03] | 1 (ref.) | 40.83  [36.72-44.95] | 1 (ref.) |
| <£50,000 | 55.74  [52.56-58.93] | 0.93  [0.65-1.34] | 80.00  [77.44-82.56] | 0.41  [0.12-1.41] | 60.21  [57.08-63.35] | 0.83  [0.55-1.26] | 44.47  [41.29-47.65] | 1.06  [0.71-1.58] |
| Prefer not to say | 62.60  [54.20-70.99] | 1.62  [0.76-3.45] | 82.44  [75.84-89.04] | 0.85  [0.09-8.24] | 63.36  [55.00-71.72] | 1.14  [0.51-2.51] | 56.49  [47.89-65.09] | 1.65  [0.79-3.47] |
| Smoking status: Smoker | 53.69  [46.78-60.61] | 1 (ref.) | 78.33  [72.61-84.04] | 1 (ref.) | 55.67  [48.77-62.56] | 1 (ref.) | 42.36  [35.51-49.22] | 1 (ref.) |
| Non-smoker | 56.52  [53.94-59.10] | 1.41  [0.83-2.38] | 81.82  [79.81-83.83] | 2.97  [0.93-9.48] | 60.25  [57.70-62.80] | 1.22  [0.67-2.23] | 44.47  [41.88-47.06] | 1.53  [0.86-2.73] |
| BMI: other | 56.27  [52.55-59.99] |  | 83.67  [80.90-86.45] | 1 (ref.) | 57.87  [54.17-61.58] | 1 (ref.) | 39.94  [36.27-43.62] | 1 (ref.) |
| Obese/overweight | 56.15  [52.71-59.58] | 1.14  [0.81-1.59] | 79.75  [76.97-82.53] | 0.97  [0.36-2.65] | 61.99  [58.63-65.35] | 1.16  [0.79-1.70] | 46.96  [43.50-50.41] | **1.63****  **[1.13-2.34]** |
| Health problems: Yes | 58.97  [55.26-62.68] | 1 (ref.) | 80.44  [77.45-83.43] | 1 (ref.) | 65.74  [62.16-69.31] | 1 (ref.) | 46.18  [42.42-49.93] | 1 (ref.) |
| No | 53.83  [50.61-57.04] | 0.89  [0.62-1.26] | 82.09  [79.62-84.57] | 0.81  [0.28-2.34] | 55.23  [52.02-58.44] | 0.67  [0.45-1.01] | 42.61  [39.42-45.80] | 1.19  [0.82-1.73] |
| Perceived high risk of Covid-19: yes | 64.22  [59.00-69.44] | 1 (ref.) | 80.54  [78.38-82.70] | 1 (ref.) | 80.12  [75.77-84.47] | 1 (ref.) | 54.43  [49.01-59.86] | 1 (ref.) |
| No | 54.13  [51.41-56.85] | **0.52 ****  **[0.32-0.85]** | 84.71  [80.79-88.63] | 1.25  [0.36-4.41] | 54.52  [51.80-57.23] | **0.26*****  **[0.13-0.52]** | 41.62  [38.93-44.31] | **0.36*****  **[0.21-0.61]** |
| Diagnosed/  suspected Covid-19: yes | 53.62  [47.70-59.54] | 1 (ref.) | 75.72  [70.63-80.81] | 1 (ref.) | 53.62  [47.70-59.54] | 1 (ref.) | 43.12  [37.24-49.00] | 1 (ref.) |
| No | 56.69  [54.04-59.34] | 0.84  [0.53-1.32] | 82.54  [80.51-84.57] | 0.88  [0.24-3.18] | 60.92  [58.31-63.53] | 0.86  [0.52-1.42] | 44.43  [41.77-47.09] | 0.87  [0.54-1.41] |
|  | **M (SD)** | **aOR**  **[95% CI]** | **M (SD)** | **aOR**  **[95% CI]** | **M (SD)** | **aOR**  **[95% CI]** | **M (SD)** | **aOR**  **[95% CI]** |
| Age | 52.88 (13.32) | 1.06  [0.94-1.20] | 51.73 (14.22) | 0.88  [0.59-1.31] | 54.07(13.05) | **1.29*****  **[1.12-1.48]** | 52.90(13.90) | 1.08  [0.95-1.24] |
| Quality of life | 3.62 (0.75) | 1.02  [0.83-1.26] | 3.62 (0.78) | 1.59  [0.88-2.86] | 3.61 (0.78) | 0.81  [0.64-1.03] | 3.63 (0.76) | 1.04  [0.83-1.30] |
| Experience of social distancing | 52.63 (22.35) | **1.10***  **[1.02-1.19]** | 51.67 (21.68) | 1.19  [0.95-1.48] | 53.53 (22.04) | **1.22*****  **[1.11-1.33]** | 52.11 (21.74) | 1.01  [0.93-1.10] |

Reference category: never adhere, age presented in decades, quality of life presented in tens, *p<0.05, **p<0.01, ***p<0.001, CI=confidence interval, aOR=adjusted odds ratio, M=mean, SD=Standard deviation.
